# Digital Resilience Biomarkers for Personalized Health Maintenance and Disease Prevention

**DOI:** 10.3389/fdgth.2020.614670

**Published:** 2021-01-22

**Authors:** Willem van den Brink, Robbert Bloem, Adithya Ananth, Thiru Kanagasabapathi, Arjen Amelink, Jildau Bouwman, Gerwin Gelinck, Sjaak van Veen, Andre Boorsma, Suzan Wopereis

**Affiliations:** ^1^Department of Microbiology and Systems Biology, Netherlands Organization for Applied Scientific Research (TNO), Zeist, Netherlands; ^2^Department of Environmental Modeling Sensing and Analysis, Netherlands Organization for Applied Scientific Research (TNO), Utrecht, Netherlands; ^3^Department of Optics, Netherlands Organization for Applied Scientific Research (TNO), Delft, Netherlands; ^4^Holst Center, Netherlands Organization for Applied Scientific Research (TNO), Eindhoven, Netherlands

**Keywords:** digital biomarker, prevention, lifestyle intervention, optical sensing, wearable sensors, resilience

## Abstract

Health maintenance and disease prevention strategies become increasingly prioritized with increasing health and economic burden of chronic, lifestyle-related diseases. A key element in these strategies is the empowerment of individuals to control their health. Self-measurement plays an essential role in achieving such empowerment. Digital measurements have the advantage of being measured non-invasively, passively, continuously, and in a real-world context. An important question is whether such measurement can sensitively measure subtle disbalances in the progression toward disease, as well as the subtle effects of, for example, nutritional improvement. The concept of resilience biomarkers, defined as the dynamic evaluation of the biological response to an external challenge, has been identified as a viable strategy to measure these subtle effects. In this review, we explore the potential of integrating this concept with digital physiological measurements to come to digital resilience biomarkers. Additionally, we discuss the potential of wearable, non-invasive, and continuous measurement of molecular biomarkers. These types of innovative measurements may, in the future, also serve as a digital resilience biomarker to provide even more insight into the personal biological dynamics of an individual. Altogether, digital resilience biomarkers are envisioned to allow for the measurement of subtle effects of health maintenance and disease prevention strategies in a real-world context and thereby give personalized feedback to improve health.

## Measurement in Health Maintenance and Disease Prevention

The majority of global deaths and health-related financial burden comes from chronic, lifestyle-related diseases, including obesity, type 2 diabetes, and cardiovascular disease (1). Health maintenance and disease prevention are, therefore, increasingly prioritized (2). Moreover, as has been highlighted during the coronavirus disease 2019 (COVID-19) pandemic, good health is important for combatting acute infections (3). To optimally exploit the potential of health maintenance and disease prevention strategies, individuals must be empowered to control their health. A core element in health empowerment is the ability to self-measure as guidance for personal health interventions (4). Unfortunately, traditional health measurements based on, for example, blood sampling or imaging are often not developed for application in a home setting and therefore less suitable for self-measurement. Limitations include their invasive nature, the lack of ecological validity, the need for trained personnel, and the high costs. Furthermore, although self-measurement tools exist, such as finger prick glucose, cholesterol, or blood pressure, these measurements are typically episodic, limiting continuous health insight and management.

Digital health solutions are promising in empowering individuals to control their health, although most developments have been focusing on disease management rather than prevention (5). Indeed, digital measurements may be passively collected in a free-living, at-home setting by anyone wearing a digital device. Digital tools such as smartwatches, smartphones, and other wearable devices are now able to measure essential indicators of health status including vital signs, skin temperature, sleep, and activity patterns (6–8). At the same time, developments of a novel type of continuous, non-invasive, and wearable molecular sensing technologies are ongoing, for example, the non-invasive quantification of biomolecules in sweat, saliva, and other body fluids (9–11). These types of measurements may eventually enrich the current abilities of digital measurements.

With the development of novel (digital) biomarkers, it is important to start with asking what health aspect is meaningful and relevant to the end-user (e.g., patient, consumer) (12). In the context of personalized health maintenance and disease prevention, it is relevant to be able to measure an intervention effect, for example, dietary change, exercise, stress coaching, or nutritional supplementation. While for some of these types of interventions, there may be clear effects on specific variables (e.g., bodyweight reduction with a weight loss program), the effects of most interventions are subtle and focused on the long term (e.g., increasing vegetable intake to improve immune health). Indeed, the goal of especially health maintenance, but also disease prevention, is to restore subtle disbalances in the biological system. To measure these subtle effects, next-generation resilience biomarkers have been proposed for the evaluation of small but relevant effects of health maintenance and disease prevention strategies (13). The foundation of these novel-type biomarkers is based on a recent definition of health by Huber et al. as “the ability to adapt and self-manage in the face of social, physical, and emotional challenges” (14). In line with this, resilience biomarkers are based on the measurement of the biological response to a specific challenge and have also been referred to as biomarkers of phenotypic flexibility (13, 15–18). Since wearable digital measurements are typically measured with a high time resolution (seconds to minutes), they are perfectly suited for the measurement of resilience as the continuous response to daily challenges such as stress, food, activity.

This review first elaborates on the concept of resilience biomarkers to subsequently explore its integration with current and future digital measurement approaches to come to a novel concept of digital resilience biomarkers for the evaluation of health maintenance and disease prevention.

## Resilience Biomarkers

**Figure 1:**
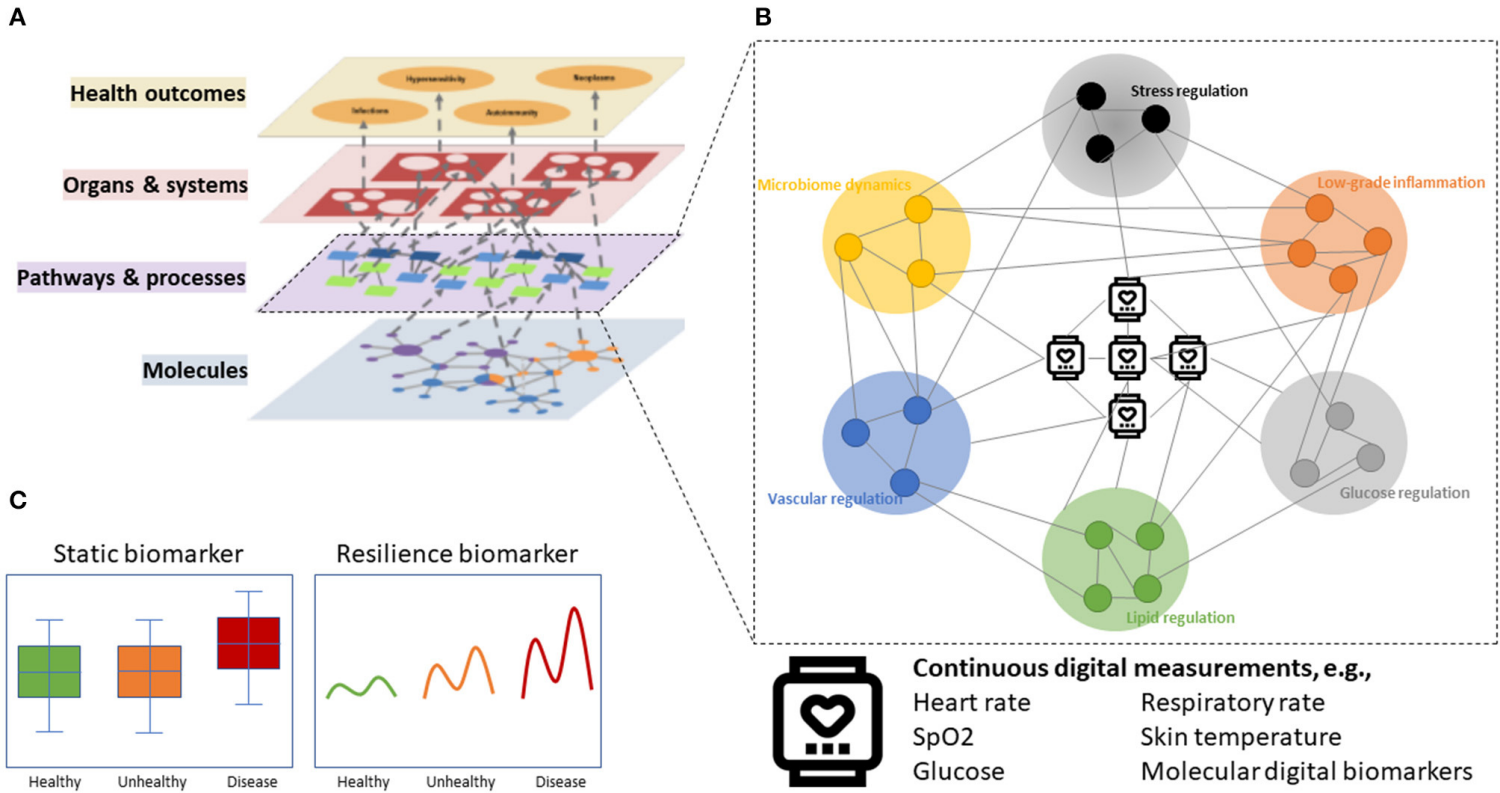
Conceptualization of digital resilience biomarkers. **(A)** The multilayer model of health with complex interactions across hierarchical layers of biological organization. **(B)** Digital sensors can be connected to molecules or biological processes of, e.g., glucose regulation, lipid regulation, vascular regulation, microbiome dynamics, stress regulation, and low-grade inflammation to come to a digital resilience biomarker. **(C)** Resilience markers are more sensitive to early and subtle derailments of biological system before disease manifests.

To evaluate the effect of personalized health maintenance and disease prevention strategies on “the ability to adapt and self-manage,” it is important to understand the dynamic interaction between the biological system and the continuously changing external influences of food, stress, activity, and other factors. A biological system is characterized by a complex, interactive network of regulatory processes that operate over multiple time scales and several layers. These layers can be defined by layers of molecules, pathways and processes, organs and systems, and health outcomes to evaluate the propagation of nutritional and other effects through the biological system (Figure 1A) (16, 19). The ability to adapt reduces with age and toward disease, starting at the bottom layers with molecules, pathways, and processes, eventually resulting in functional decline at the level of health outcomes. For instance, as an isolated example, overnutrition (molecules) causes insulin resistance (process), which in turn can cause accumulation of hepatic triglycerides leading to hepatic steatosis (organ), eventually causing fatty liver disease (health outcome) (20).

**Figure 2:**
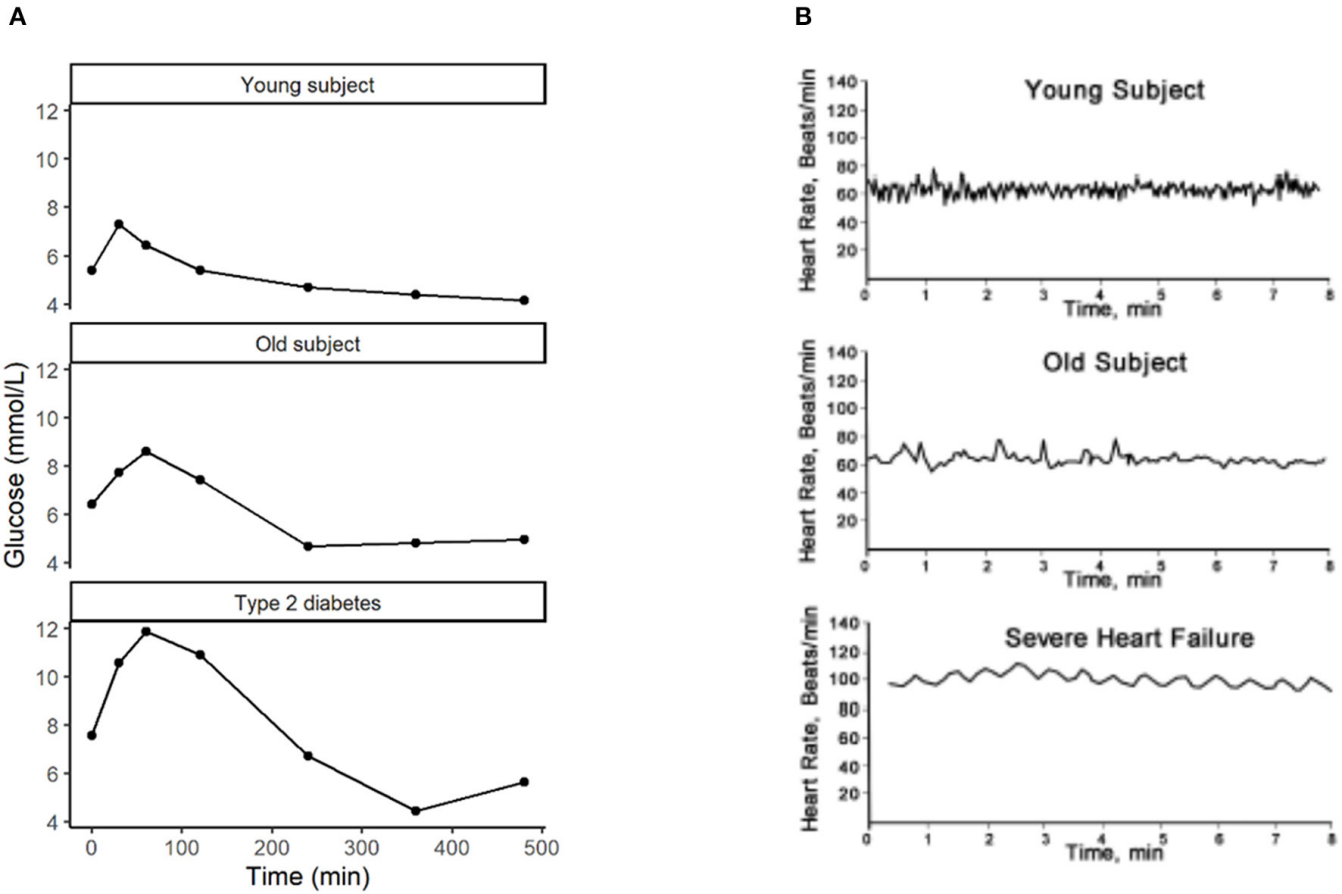
Measures of resilience with **(A)** the increased response of glucose to a high caloric milkshake with age and disease [data from studies described by Wopereis et al. (21) and van den Broek et al. (22)] and **(B)** the decrease of heart rate variability with age and disease [reprinted with permission from Sturmberg et al. (23)].

A resilience biomarker aims to measure “the ability to adapt” that is reduced with age and disease (Figures 1B, 2). The generally accepted definition of a biomarker is “a defined characteristic that is measured as an indicator of normal biological processes, pathogenic processes, or responses to an exposure or intervention, including therapeutic interventions” (24). In 2016, an Food and Drug Administration–National Institutes of Health (FDA-NIH) working group identified seven biomarker subtypes being diagnostic biomarker, monitoring biomarker, pharmacodynamic/response biomarker, predictive biomarker, prognostic biomarker, safety biomarker, and susceptibility/risk biomarker. Definitions of these subtypes are given in Table 1 (24). With none of these biomarkers addressing “the ability to adapt” as a concept with particular utility in the health maintenance and disease prevention domain, we have highlighted the resilience biomarker as a separate subcategory of a diagnostic, monitoring, or response biomarker (Table 1).

**Table 1:** Definitions, non-digital, and digital examples for eight biomarker types.

**Type**	**Definition^**a**^**	**Non-digital example**	**Digital example**
Resilience biomarker^b^	A specific type of a diagnostic, monitoring, or response biomarker measured as the response to an external challenge to reflect the resilience of the biological system	Cytokine response to the PhenFlex challenge indicates low inflammatory resilience in metabolically compromised individuals (25)	Continuous glucose variation is higher in prediabetic and diabetic individuals with glucose dysregulation (26)
Diagnostic biomarker	A biomarker used to detect or confirm presence of a health status, disease or condition of interest or to identify individuals with a health or disease subtype.	Increased fasting plasma glucose or hemoglobin A1c indicate the presence of type 2 diabetes	Atrial fibrillation is diagnosed^c^ using photoplethysmography in a smart watch (27)
Monitoring biomarker	A biomarker measured serially for assessing status of health or a disease or medical condition or for evidence of exposure to (or effect of) a medical product, a lifestyle change, or an environmental agent	Regular glucose finger pricks are used to manage insulin administration in type 1 diabetes patients	Calibration-free continuous glucose monitoring is used for monitoring type 2 diabetes patients (28)
Response biomarker	A biomarker used to show that a biological response has occurred in an individual who has been exposed to a medical product, a lifestyle change, or an environmental agent	Serum LDL cholesterol reduction with cholesterol lowering agents or lifestyle changes	Hyperglycemic peak reduction with exercise as an add-on intervention in type 2 diabetes patients using metformin (29). Hyperglycemic peaks were measured with continuous glucose monitoring
Predictive biomarker	A biomarker used to identify individuals who are more likely than similar individuals without the biomarker to experience a favorable or unfavorable effect from exposure to a medical product, a lifestyle change, or an environmental agent	Individuals with insulin resistance in the muscle benefit from a Mediterranean diet, whereas individuals with insulin resistance in the liver mostly benefit from a low-fat diet (30)	Wearable heart rate, skin conductance, skin temperature, and activity patterns were used to define digital phenotypes characterized by poor health indicators and high depression, anxiety and stress scores (31)
Prognostic biomarker	A biomarker used to identify likelihood of a clinical event, disease recurrence or progression in patients who have the disease or medical condition of interest.	Augmented C-reactive protein (CRP) levels indicate increased likelihood of recurrent artery disease events in patients with unstable angina	Spatial memory, prospective memory, executive function, and psychomotor processing speed were assessed with a smartphone app to define a prognostic biomarker for progression to dementia in people with mild cognitive impairment (32)
Safety biomarker	A biomarker measured before or after an exposure to a medical product, a lifestyle change, or an environmental agent to indicate the likelihood, presence, or extent of toxicity as an adverse effect	Increased levels of plasma creatinine phosphokinase indicate statin intolerance in the muscles (33)	QT prolongation, a cardiac safety biomarker, was reliably identified using a wearable ECG monitoring system (34)
Susceptibility/risk biomarker	A biomarker that indicates the potential for developing a disease or medical condition in an individual who does not currently have clinically apparent disease or the medical condition	High CRP levels indicate greater likelihood of developing incident coronary disease (35)	Autonomic imbalance, measured by reduced heart rate variability, may serve as a risk factor for cardiovascular disease (36)

a *Definitions are adapted from BEST glossary also include health status and lifestyle interventions (24)*;

b *Added to the list to highlight the specific characteristic of a resilience biomarker*;

c *At the moment, this is not a medical diagnosis*.

There are two approaches for constructing a resilience biomarker as the dynamical response to external perturbations to the biological system. The first approach is based on the time-resolved biomarker response to a standardized challenge (13). Examples of standardized challenges have been developed for multiple health domains and include the cardiopulmonary exercise test (37), Trier social stress test (38), lipopolysaccharide challenge (to quantify immune resilience) (39), oral glucose tolerance test (40), and mixed-meal challenges (15). Healthy individuals adequately handle a challenge test through regulatory mechanisms of adaptation, showing optimal resilience. The loss of resilience is reflected in an impaired response to the challenge (21, 22, 41, 42). Figure 2A presents an example of the oral glucose tolerance test as the measurement of blood glucose dynamics upon ingestion of 75 g of water-dissolved sugar within 5 min. The effect of aging, and even more so of disease, can be observed in the higher glucose concentrations in response to the same challenge. In other words, the regulatory mechanisms of glucose uptake are impaired in older individuals and type 2 diabetes patients.

To further understand the dynamical interaction between the biological system and external perturbations, it is important to measure the integrated biological response to a challenge. Therefore, more complex resilience biomarkers have been developed by evaluating the dynamic response of multiple biomarkers to a single challenge test, for example, the PhenFlex challenge (21). This is a standardized high-caloric mixed meal challenge that has been proven to perturb multiple biological processes, including glucose metabolism, lipid metabolism, amino acid metabolism and vitamins, metabolic stress, and low-grade inflammation (21). Based on the differential biomarker responses to the PhenFlex challenge in individuals along a health spectrum ranging from healthy to unhealthy, several resilience biomarkers were developed associated with these biological processes (22). Not only have these resilience biomarkers allowed for the quantification of health in the non-disease range, but they have also been shown to quantify the effect of subtle nutritional interventions (25, 43).

A second way of constructing a resilience biomarker is to evaluate a continuous biological pattern without using an explicit standardized challenge test. For instance, a continuous electrocardiogram can be used to determine indices of heart rate variability as a reflection of the autonomic nervous system function (44). Indeed, heart rate patterns do change with age and disease, as can be observed in Figure 2B. In fact, in this example, the mathematical complexity (e.g., sample entropy) of the continuous pattern is reduced and can be used as a measure of biological resilience sensitive to age and disease (23, 45). Since this approach of constructing resilience biomarkers often involves a digital measurement, further examples will be discussed below.

## Digital Resilience Biomarkers

The measurement of a challenge–response involves time-resolved sampling. The relative burden for the collection of this type of resilience biomarkers, besides being exposed to a standardized perturbation, is mostly thus higher than for a static biomarker. As an example, the measurement of metabolic resilience based on traditional measurement methods includes five blood samples throughout 4 h (22). Moreover, such measurement only gives insight into the resilience at a specific moment. Digital biomarkers have the advantage of being measured non-invasively and continuously. They can be defined as “biomarkers collected by a wearable or portable system of sensors, electronics, and algorithms that generate a long-term, real-time digital signal to enable frequent, non-invasive monitoring under daily life conditions” [adapted from (6)]. This definition of a digital biomarker includes measurements from smartwatches, smart textiles, smart scales, and handheld devices for real-time health monitoring by consumers but excludes clinical-grade devices that need operation by a professional. For example, an at-home real-time temperature measurement falls under this definition but not a lab-based hair cortisol measurement. During the last decade, the number of digital biomarkers has grown rapidly. Examples of digital biomarker applications in a general population include photoplethysmography for the detection of irregular pulse rate to predict atrial fibrillation (27) and accelerometry for measurement of sleep duration (46). Other examples, focused on a specific disease population, are the measurement of accelerometry and gyroscopy for gait freezing in Parkinson's disease (47); the use of accelerometry and skin conductance to predict epileptic seizures (48); wearable electroencephalogram, heart rate variability, and skin conductance for mood tracking (49); photoplethysmography for detecting diabetes (50); or a combination of pulse rate, skin conductance, skin temperature, and oxygen saturation for telemonitoring of chronic obstructive pulmonary disease (COPD) patients (51).

Being measured in time series, digital biomarkers are well-suited for constructing a resilience biomarker based on a continuous biological pattern. As already mentioned, heart rate variability is a resilience biomarker reflecting autonomous system function related to multiple biological functions (52, 53), including psychological stress (54), cardiovascular health (36, 55), and inflammation (56, 57). Other examples include variability of blood pressure (58–60), gait (61, 62), and glucose (26). Blood pressure variability has been associated with all-cause mortality (59), cardiovascular events (59), and progression of Alzheimer's disease (60). Gait variability has been associated with the risk of falling in neurodegenerative disorders (61), although it seems not to change with age (62). Glucose variability was associated with glucose dysregulation (26). While these examples focus on a digital resilience biomarker as a complexity measure of a digital time-series pattern, they can also be defined as the digital biomarker responses after a standardized challenge test or as the continuous response upon daily challenges, such as food intake and activity (63).

By selecting digital measurements based on their ability to reflect a specific biological process, a digital resilience biomarker can be constructed (as conceptualized in Figure 1C) to evaluate subtle changes in the biological system. Here, we describe several examples of biological processes relevant to health maintenance and disease prevention that may be measured using digital resilience biomarkers. These biological processes can be digitally quantified alone, or in combination, to provide an integrated measurement of diverse health aspects. For illustration purposes of future potential, not all examples will strictly follow the abovementioned definition of a digital biomarker. Some examples of measurement methods are slightly invasive (e.g., continuous glucose monitors) or currently only available as a clinical-grade methodology (e.g., ambulatory blood pressure monitoring). In addition, it should be noted that developing digital resilience biomarkers involves algorithm development, but a discussion on specific data science methods is not within the scope of this review.

### Digital Resilience Biomarkers of Vascular Regulation

Dysregulated vascular homeostasis is an important determinant in several metabolic diseases, including cardiovascular disease, metabolic syndrome, and type 2 diabetes, and is associated with oxidative stress, chronic inflammation, insulin resistance, and lipid dysregulation (64). It now becomes possible to monitor vascular function by looking at continuous patterns from wrist-worn tonometry (65), photoplethysmography (66), or ambulatory blood pressure variability (67) as a digital resilience biomarker. Blood pressure variability has been associated with cardiovascular disease outcome (59). In particular, the short-term variability (beat-to-beat and 24 h) is of interest, being related to arterial compliance or arterial elasticity (58), although it has been questioned whether the value of blood pressure variability outperforms that of absolute blood pressure for the prognosis of cardiovascular outcome (68).

### Digital Resilience Biomarkers of Mental Stress Regulation

Mental stress is an important driver of, among others, neurodegenerative, mental, cardiovascular, metabolic, and inflammatory diseases (4, 18, 69). Whether sources of stress have a negative influence on long-term health outcomes is dependent on the mental resilience of an individual. Indeed, the physiological response, including heart rate, to the Trier Social Stress Test indicates the resilience to stress (38). Not only heart rate, heart rate variability, skin conductance, and skin temperature but also voice perturbations have been used in response to a stress test for the quantification of stress under laboratory conditions (70–72). Not all of these variables may be reliably measured with consumer-grade wearables under non-stationary daily life conditions, given their sensitivity to motion artifacts (73). However, large-scale monitoring of the electrocardiogram, skin conductance, and skin temperature under daily life circumstances revealed blunted physiological stress responses associated with poor health, high depression, high anxiety, and high levels of stress (31).

### Digital Resilience Biomarkers of Chronic Low-Grade Inflammation

Chronic low-grade inflammation plays a central role in many lifestyle-related chronic disorders including cardiovascular disease and metabolic syndrome (43). Recently, the cytokine response to the PhenFlex challenge was used to define a non-digital resilience biomarker of chronic low-grade inflammation (25). Interestingly, heart rate variability (57, 74), and blood oxygen saturation (690 may act as indirect digital biomarkers of inflammation. Indeed, heart rate variability has consistently been associated with C-reactive protein and other inflammatory markers (57). In addition, skin temperature measured by a wearable may be associated with systemic inflammation, although this association was not consistent, possibly explained by the influence of environmental temperature on skin temperature (75).

### Digital Resilience Biomarkers of Host–Microbiome Dynamics

The host–microbiome dynamics, greatly determined by dietary intake, is an important mediator between diet and health through inflammatory, metabolic, and neural processes (76, 77). Hydrogen breath testing upon a lactose challenge is a proven technique to determine lactose intolerance, based on defect microbial carbohydrate fermentation. Processes of microbial fermentation produce volatiles such as hydrogen, methane, and hydrogen sulfide, among other volatiles, that can be measured in breath by portable eNose technology (78–80). Although only starting to emerge, devices for measurement of these compounds in breath are becoming small and consumer grade, enabling their utilization in a daily life (81), holding promise for these biomarkers becoming a digital resilience biomarker for microbiome dynamics.

### Digital Resilience Biomarkers of Lipid Regulation

Lipids serve multiple essential functions in the body, including energy storage, acting as structural components of cells, and signaling. Biological processes underlying lipid regulation include lipid digestion, transport, storage, and metabolism. Currently, there are no digital biomarkers known for these markers, although early developments are being made for wearable measurement of cholesterol (82). In addition, intra-abdominal fat spectroscopy, associated with lipid regulation and inflammation, is likely to become possible in the near future (83). Other than that, the autonomic imbalance is associated with metabolic syndrome and its components, including cholesterol and triglycerides (84, 85), indicating that heart rate variability may serve as an indirect reflection of lipid dysregulation.

### Digital Resilience Biomarkers of Glucose Regulation

Glucose regulation is controlled by a series of biological processes, including insulin sensitivity, β-cell function, and gluconeogenesis. As a digital biomarker, glucose can be reliably measured in the interstitial fluid of the skin by minimally invasive continuous glucose monitoring for up to 14 days without the need for invasive calibration (28, 86). The oral glucose tolerance test response, measured by a continuous glucose monitor, was recently used to evaluate the postprandial responses to food (63). Continuous glucose monitoring was also used to calculate glucose variability as a measure of glucose dysregulation (26). Heart rate variability was associated with measures of systemic insulin sensitivity (75, 87), showing prognostic value for predicting 5-year insulin sensitivity (88). Given its generic character, the specificity for measuring heart rate variability as a proxy for glucose dysregulation is low. However, this may be improved by perturbing glucose regulation with an oral glucose tolerance test (89). This approach, heart rate variability response to an oral glucose tolerance test during pregnancy, may even allow for indirect determination of fetal insulin sensitivity using fetal magnetocardiography (90).

## Molecular Digital Biomarkers

The above-described digital resilience biomarkers are mostly based on measurements of vital signs such as heart rate, blood pressure, or oxygen saturation. While a few examples of minimally or non-invasive measurements of specific molecular biomarkers (subcutaneous glucose, breath volatiles) were discussed, this comprises a much larger novel area with future potential for novel, specific molecules measured as digital biomarker (9–11). Given the fact that these technologies allow the continuous measurement of molecular markers, they can also be used to define novel digital resilience biomarkers. Currently, many of these molecular digital biomarkers are at the stage of evaluating and demonstrating the performance of the sensor technology. Molecular digital biomarkers can be measured transcutaneous in blood or tissue or a non-invasively accessible biofluid such as sweat, tears, or saliva (Table 2).

**Table 2:** Digital biomarker characteristics, challenges and sensing principles of molecular digital biomarkers in non-invasive biofluids^a^.

**Sensing modality**	**Digital biomarker characteristics (current state of the art)**				**Technical challenges^**b**^**	**Main sensing principle**	**(Potential) molecular markers^**c**^**
	**Non-invasive**	**Real-time**	**Continuous**	**Wearable**			
Sweat	Yes, although some cases involve invasive stimulation of sweat production by iontophoresis	Yes	Yes, days to weeks	Yes, platforms include wristbands, tattoos, patches and textiles	- Low samples volumes - Low biomarker concentrations - Contamination of consecutive sweat samples - Artifacts from sweat rate, temperature, pH - Biomolecule distribution from blood to sweat (including time-lag)	Selector-transducer (electrochemical, optical)	Metabolites (e.g., glucose, lactate, ethanol), electrolytes (e.g., pH, Na^+^, Cl^−^), heavy metals
Interstitial fluid	Currently minimally invasive due to subcutaneous insertion of cannula or interstitial fluid (ISF) collection by reverse iontophoresis	Yes	Yes, days to weeks	Yes, platforms include patches and wristbands	- Interference from sweating (with reverse iontophoresis) - Low sample volumes - Skin irritation due to ISF extraction - Biomolecule distribution from blood to interstitial fluid (including time-lag)	Selector-transducer (electrochemical, optical)	Metabolites (e.g., glucose, urea, pharmaceuticals)
Tears	Yes	Yes	Yes, days to weeks	Yes, platform used is a contact lens	- Transparency - Biocompatibility - Application in humans - Biomolecule distribution from blood to tears (including time-lag)	Selector-transducer (electrochemical, optical)	Metabolites (e.g., glucose, lactate)
Saliva	Yes	Yes	Yes, days to weeks	Yes, platforms include tooth enamel, mouthguard and pacifier	- Contamination with food residues, bacteria, etc. - Mechanical stress on sensor from mouth movements - Biocompatibility and user comfort - Biomolecule distribution from blood to saliva (including time-lag)	Selector-transducer (electrochemical, optical)	Metabolites (e.g., cortisol, glucose, lactate, uric acid)
Breath	Yes	Yes	Currently limited, due to non-wearable platforms	No, mostly portable, although a wristband has been developed requiring active breathing onto the sensor	- No current wearable applications - Contamination from ambient air - Artifacts from airflow, humidity, ingested materials and temperature	Selector-transducer (electrochemical, optical), spectroscopy	Metabolites (e.g., hydrogen, methane, sulfate)
Transcutaneous (tissue, blood)	Yes	Yes	Yes, months to years	Yes, depending on the spectroscopic method.	- Motion artifacts - Signal-to-noise - Need for frequent calibration - Often indirect measurement, which is sensitive to confounders	Spectroscopy	Metabolites (e.g., oxygen saturation, fat, water, NADH, FAD, bilirubin), proteins (e.g., advanced glycated end products)

a *This table is a compilation of information from multiple sources. Worthwhile reviews on the sensor technologies, molecular markers, and the related challenges can be found in (9, 10, 91)*.

b *Not including the general challenges of bio-sensing (e.g., stability, sensitivity, etc.), energy supply, wireless communication, material size and rigidity, and data analytics and security (91)*.

c *The list of potential biomarkers is broader if purely local biomolecules (e.g., proteins, peptides, bacteria) are also considered. The examples in this table are limited to those with potential to distribute from the systemic circulation to the biofluid of interest*.

### Non-invasive Sampling of Biofluids for Molecular Digital Biomarkers

One of the most explored non-invasive biofluids for continuous monitoring is sweat (10). Eccrine sweat glands are located over nearly the whole body surface, and sweat contains multiple biomolecules that are generated locally or transported from the systemic circulation via diffusion or active transporters (92). Examples of biomarkers that are being explored for continuous and real-time analysis in eccrine sweat include metabolites (e.g., glucose, lactate, cortisol, uric acid, alcohol), electrolytes (e.g., chloride, sodium, potassium, pH), and metal traces (e.g., zinc, iron, copper) (9, 10). Furthermore, interstitial fluid, although currently collected in a minimally invasive manner, is well-known for the continuous glucose monitoring devices on the market, but it contains many more biomolecules. Interstitial fluid can be collected via a subcutaneous cannula, or via reverse iontophoresis (93), to allow for contact between the sensor and the analyte. An interesting development is the application of pain-free microneedles for multiplex monitoring of biomolecules in interstitial fluid (94), as was shown for glucose, lactate, and pH (95). In addition, tear fluid contains metabolites, electrolytes, and proteins, some of them reflecting systemic concentrations. Although, currently, most studies have been performed in animals, sensors integrated into contact lenses have been developed to monitor biomolecules in tears as digital biomarkers, including glucose and lactate (96, 97). Saliva is another attractive biofluid for molecular digital biomarkers, containing metabolites (e.g., glucose, lactate, cortisol), enzymes (e.g., alpha-amylase), and antibodies (e.g., IgA, IgG) (98). Saliva monitoring has been accomplished by sensor integration in, for example, a tooth enamel for bacteria detection (99), a mouthguard for glucose and nitrite monitoring (100), and even a pacifier for glucose monitoring (101). In addition, exhaled breath contains many volatile organic compounds that have been shown predictive of several diseases, including diabetes (102). Combined with a lactose challenge, breath hydrogen testing is the gold standard for detecting lactose intolerance, often also combined with other breath volatiles such as carbon dioxide and methane. This test is now also available in a portable, consumer-grade device, although further optimization is needed to improve the accuracy of the measurement (81). Finally, non-invasive monitoring of biomolecules in blood and tissue is possible with spectroscopic techniques (103). A well-known example is pulse oximetry, available as a medical device, used for the detection of peripheral oxygen saturation in the fingertip (103).

### Sensing Principles for Molecular Digital Biomarkers

Four different sensing principles are used for biomonitoring in general: electrophysiological monitoring (e.g., electrocardiogram), acoustic monitoring (e.g., ultrasound), selector-transducer monitoring (mostly electrochemical sensors), and optical monitoring (e.g., pulse oximetry for oxygen saturation and heart rate). The sensing principles that are most relevant for non-invasive biofluid and transcutaneous biomolecule detection are selector-transducer and optical spectroscopy-based detection methods. Table 3 provides an overview of specific sensor types based on these principles that will be discussed below.

**Table 3:** Sensor types and related sensing principles for wearable, non-invasive, continuous molecular digital biomarkers.

**Sensor type**	**Sensing principle^**a**^**
**Selector-transducer**	
Potentiometric sensors	An ionophore binding specific ions (e.g., Na^+^, K^+^, etc.) combined with a transducer that senses the voltage differences with a reference electrode
Amperometric sensors	An enzyme catalyzing the target metabolite (e.g., glucose, lactate, etc.) combined with a transducer that senses the change in the electrical current at an electrode
Conductometric sensors	An enzyme catalyzing the target analyte combined with a transducer that senses the changes in ionic conductance
Colorimetric sensors	A sensor that changes color upon binding with a specific metabolite (e.g., glucose, lactate) or an electrolyte (e.g., Na^+^, Cl^−^)
Fluorometric sensors	A sensor that changes fluorescent properties upon interaction with a specific metabolite (e.g., glucose, lactate, O2) or an electrolyte (e.g., Na^+^, Cl^−^)
**Spectroscopy**	
Transmission spectroscopy	Transmission of light at a specific wavelength (ultraviolet, visible, near-infrared, infrared) through a sample to measure the absorption, which is proportional to the number of molecules of interest. A well-known example is Fourier Transform Infrared (FTIR) spectroscopy
Reflectance spectroscopy	Reflectance of light at a specific wavelength (ultraviolet, visible, infrared, near-infrared) in a sample to measure the absorption, which is proportional to the number of molecules of interest
Photoplethysmography	A specific form of transmission or reflectance spectroscopy to detect volume changes in peripheral blood vessels as a measure of heart rate and other cardiovascular variables
Photoacoustic spectroscopy	Energy gained by light absorption is released as heat in a gas chamber or tissue to form a pressure wave measured as sound, mostly applied for breath analysis
Photoluminescence (fluorescence) spectroscopy	Energy gained by light absorption is released as light with longer wavelength due to energy loss to thermal energy
Raman spectroscopy	Energy gained by light absorption is released as light, with a slightly different energy because of interactions with vibrational modes in the molecules

a *Definitions of the sensing principles are based on those described in dedicated reviews (9, 10, 91, 103)*.

The selector-transducer principle is based on a selector (e.g., an enzyme) that selectively and with high sensitivity interacts with the biomarker of interest to produce a signal that is related to the concentration of the biomolecule of interest (104, 105). For a digital biomarker, this interaction needs to be fast and reversible to allow for continuous, real-time monitoring. The interaction between the biomarker and the selector is translated into a signal by a transducer function and mostly also an indicator. The electrochemical selector-transducer principle is one of the most widely used for biomarker detection, although also acoustic, piezoelectric, and optical (colorimetric, fluorometric) transducer functions have been applied (104, 105). Both the selector as well as the indicator are incorporated in a polymeric or ceramic coating. Examples are the electrochemical and optochemical detection of glucose using the enzymes glucose oxidase or hexokinase as selectors. Glucose oxidase produces H_2_O_2_, causing a change in electrical current measured by amperometry. Hexokinase produces NADH that influences the absorption of light that can be measured by photometry. This principle has been applied in sweat, tear fluid, saliva, interstitial fluid, etc. Similarly, many other biomolecules can be measured using a specific selector-transducer principle, including metabolites (lactate, uric acid, ammonia, drugs, carbon dioxide, cortisol, sugars, etc.) and electrolytes (H^+^, Na^+^, Cl^−^, etc.) (9, 10). Larger molecules such as peptides and proteins can also be detected with these principles, although these molecules are minimally present in sweat, tears, etc. due to limited solvability and diffusion. However, since these molecules are not expected to distribute from the systemic circulation to the biofluids of interest because of their size, they are regarded as less relevant as digital resilience biomarker for systemic biological processes. Wearable, continuous electrochemical detection is only starting to emerge with current applications mostly being research-grade.

Optical methods, in the most basic form, use a light source to measure the absorption that is proportional to the number of molecules of interest. The absorption of light is based on molecular electronic or vibrational transitions that occur at specific wavelengths of light (ultraviolet, visual, infrared, etc.) and are different for every molecule. A common detection method is to measure the transmission or reflection of specific wavelengths to calculate the absorbance. A well-known example of transmission spectroscopy is the measurement of oxygen saturation (103), using two light-emitting diodes at different wavelengths, one being red (wavelength 660 nm) and the other being infrared (wavelength, 880 or 940 nm), and a photodiode for measuring the transmission through a finger or earlobe at both wavelengths. Deoxygenated and oxygenated hemoglobin have different absorption characteristics at both wavelengths, allowing for the calculation of oxygen saturation from their transmission intensities. Similarly, wearable pulse rate measurements are often based on reflectance photoplethysmography using red or green light-emitting diodes (103). Alternatively, some optical methods measure the fluorescent light that is released by a molecule after absorbing light. Raman spectroscopy is based on Raman scattering, causing a slight shift in wavelength because of the interactions with vibrational transitions in the molecules. The technology has been applied to non-invasively measure glucose, although the current evidence is based on small sample-sized and limited external validation (106). Photoacoustics measures the absorbed energy that is converted into heat causing local expansion, which generates a pressure wave in a gas chamber or tissue that can be measured as ultrasound. The technology has, for example, been used to measure ethylene, as a novel biomarker for early onset of infection (107). Photoluminescence, based on intrinsic fluorescent or phosphorescent molecular characteristics, measures the released energy as light with a longer wavelength due to energy loss to heat. These last two methods are very sensitive because the released energy has a different form with no background signal.

### Challenges With Molecular Digital Biomarkers

Wearable biomonitoring platforms have to deal with multiple challenges (Table 2). General challenges include operational challenges of pretreatment conditions, stability, sensitivity, response time, and multianalyte interference, energy supply challenges, data communication challenges, material challenges of size and rigidity, and data analytics and security challenges. These are all crucial aspects to address when developing a digital biomarker. A detailed discussion on these topics is out of the scope of this review but is provided elsewhere (91). Additionally, when non-invasively identifying digital biomarkers from accessible biofluids, reflecting systemic concentrations, the disconnection between systemic biomolecule concentrations and local concentrations may be a challenge. Transport of biomolecules, local production, external influences, and internal milieu are all factors that can affect this relation that need to be taken into account for developing a reliable sensor for molecular digital biomarker monitoring. Furthermore, more specifically, continuous sweat monitoring has to deal with low sample volumes, low biomarker concentrations, contamination of consecutive sweat samples, and artifacts from sweat rate, pH, and temperature (10). Interstitial fluid monitoring probably is the most advanced technology with the main challenges being its relative invasiveness, interference from sweating, skin irritation (both with reverse iontophoresis), and low sample volumes (10). Tear fluid monitoring must guarantee transparency and biocompatibility, and still needs a translation to humans for further validation (10). Saliva monitoring is challenged by contamination from food residues, bacteria, etc., mechanical stress from mouth movements on the sensor, and biocompatibility and user comfort (e.g., with a mouthguard) (10). Breath monitoring is challenged by contamination from ambient air and artifacts from airflow, humidity, ingested materials, and temperature (10). Transcutaneous optical methods may suffer from low sensitivity due to low signal-to-noise ratios and artifacts such as motion and ambient light. Additionally, frequent calibration may be needed in cases where there is a risk of confounding effects. Nevertheless, multiple developments are ongoing to overcome these limitations and to develop reliable at-home biomonitoring platforms that eventually allow for molecular digital health monitoring and the development of digital resilience biomarkers.

## Utilizing Digital Resilience Biomarkers for Personalized Health Maintenance and Disease Prevention

For digital resilience biomarkers to be optimally utilized, they must take an integral part in eHealth applications that focus on personalized health maintenance and disease prevention. Chronic disorders are typically complex and require a systems approach to cure or prevent them. This not only involves biological components but also behavioral and social elements, allowing tailor-made (lifestyle) interventions based on the individual needs from all domains that affect the disease (4, 8). Digital measurements can cover several of those elements enriching resilience biomarkers with static digital biomarkers to gain a more comprehensive biological measurement. Furthermore, digital measurements of behavior can include activity trackers, sleep trackers, and stress monitoring, whereas mobile phone usage and Global Positioning System (GPS) data can be used for monitoring the social context. As an example, Van Ommen et al. have convincingly argued for personalized systems interventions (i.e., dietary interventions, physical activity, medication), taking these factors into account to achieve remission of type 2 diabetes by regaining biological resilience on beta-cell function, insulin sensitivity, vascular health, and chronic low-grade inflammation (4). Similarly, from a health maintenance and disease prevention perspective, personalized systems interventions will help maintain biological resilience. Indeed, mHealth platforms such as WellDoc have shown clinical improvement of diabetes (108). Similar applications have been shown effective for diabetes prevention now (109). As we have argued in this review, digital resilience biomarkers provide an accessible way to measure the biological resilience related to glucose health, chronic low-grade inflammation, vascular health, and other biological domains.

Several of the above-described digital resilience biomarkers are based on sensor technology available in current wearable technology. The sensor data from these wearables can be evaluated in response to a challenge test or as a continuous pattern to define a digital resilience biomarker. Awaiting further validation of such digital resilience biomarkers, a relatively straightforward integration into existing mHealth platforms is possible. While the examples focus on (pre)diabetes, the idea of digital resilience biomarkers as a component of eHealth platforms will be relevant for other chronic diseases (e.g., cancer, mental disorders, neurodegenerative disease) to quantify early development or remission of the disease. In addition, it will be helpful as an instrument to monitor people at risk for developing chronic disease [e.g., those with genetic disposition or adverse childhood experiences (110)] to guide preventive interventions.

## Concluding Remarks

Digital biomarkers have gained large interest during recent years as non-invasive markers of health and disease. As they typically allow for continuous monitoring, they may also be used for the development of resilience biomarkers. These biomarkers, in contrast to static biomarkers, allow the quantification of subtle disbalances in the biological network associated with early progression toward disease. The combination of digital biomarker development with the concept of resilience provides a novel type of digital biomarkers as outlined in this review. A digital resilience biomarker is based on the dynamical interpretation of a non-invasive and continuous digital biomarker measured in daily life. Although most of the digital resilience biomarker examples described in this review come from a lab setting, these biomarkers have good potential for application in an at-home setting. Future research should focus on the validation of these biomarkers, ideally guided by a recently published framework around validity, usability, and data security (5). Furthermore, with all efforts focusing on developing wearable electronics for molecular monitoring within accessible biofluids, novel digital resilience biomarkers will become available that give mechanistic insight into biological pathways and processes concerning health status and dynamics. Development of the digital resilience biomarker concept is envisioned to eventually allow for non-invasive, continuous monitoring of personalized health maintenance and disease prevention strategies under real-world conditions. Digital resilience monitoring can be combined with personalized intervention strategies to improve individual health.

## Author Contributions

WB, AB, and SW conceived and conceptualized the manuscript. WB coordinated the collaboration. All authors wrote, reviewed, and approved the manuscript.

## Conflict of Interest

The authors declare that the research was conducted in the absence of any commercial or financial relationships that could be construed as a potential conflict of interest.
